# Update on ketosis in dairy cattle with major emphasis on subclinical ketosis and abdominal adiposity

**DOI:** 10.1002/vms3.1525

**Published:** 2024-08-30

**Authors:** Pedro Melendez, Manuel Vizcaino Serrano

**Affiliations:** ^1^ Department of Veterinary Clinical Sciences Jockey Club College of Veterinary Medicine & Life Sciences City University of Hong Kong Hong Kong SAR China

**Keywords:** abdominal fat, dairy, energy balance, inflammation, ketosis, lipolysis, milk

## Abstract

The metabolic changes that occur during the early post‐partum period in dairy cows can indeed lead to an imbalance in energy utilization, resulting in the production of excessive ketone bodies. This can have detrimental effects on the cow's health and milk production, leading to economic losses for dairy producers. The release of non‐esterified fatty acids into the blood due to increased lipolysis is a key factor in the development of ketosis. Abdominal adiposity is a key factor on these outcomes in modern dairy cows. The redirection of energy and glucose towards lactose synthesis and milk yield leaves a deficit of gluconeogenic precursors, leading to the conversion of acetyl‐CoA into ketone bodies instead of entering the Krebs cycle. These ketone bodies, including acetone, acetoacetate and β‐hydroxybutyrate, accumulate in the blood and can be detected in various bodily fluids, such as urine, blood and milk, allowing for diagnostic testing. Prevention is indeed crucial in managing ketosis in dairy cattle. Supplementation of propylene glycol in the diet or the use of monensin, either in the diet or in the form of a slow‐release bolus, can help prevent the occurrence of ketosis. However, avoiding high body condition (subcutaneous fat) and excessive abdominal adiposity during the dry period and parturition plus an adequate cow comfort are fundamental tasks to avoid ketosis and related disorders. These interventions aim to provide additional energy sources or enhance the cow's ability to utilize energy efficiently, thus reducing the reliance on excessive lipolysis and ketone body production.

## Graphical abstract

The metabolic changes that occur during the transition period of dairy cows can lead to an imbalance in energy metabolism, resulting in excessive ketone body production. They have detrimental effects on lactational performance, leading to high economic losses on dairy farms. Excessive lipolysis is a key factor in the development of ketosis; consequently, avoiding high body condition scores and excessive abdominal adiposity during the transition period, along with adequate cow comfort, are fundamental tasks to prevent ketosis and related disorders.

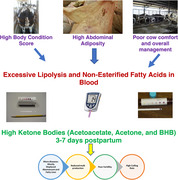

## 1 INTRODUCTION

Ketosis is a metabolic disorder that primarily affects high‐producing dairy cows. It is characterized by an increased level of ketone bodies in the bloodstream, including acetone (Ac), acetoacetate (AcAc) and β‐hydroxybutyrate (BHB). This condition can manifest in both clinical and subclinical forms. The clinical presentation often involves neurological signs like abnormal wandering, unsteady gait, bellowing and occasional aggression. It may also lead to reduced feed intake, decreased milk production and disruptions in other physiological functions (Chirivi et al., 2023; Melendez, 2017).

On the other hand, subclinical ketosis (SCK) occurs when there is an elevated concentration of ketone bodies, particularly BHB, in the blood. In this case, the signs may be less apparent, but it can still have significant effects, such as reduced fertility and increased susceptibility to secondary diseases like mastitis, metritis, fatty liver and displacement of the abomasum (DA). It is worth mentioning that milk production can be normal, reduced or even increased, depending on various factors within the herd (Melendez, 2017; Melendez & Risco, 2021).

Furthermore, cows with SCK are more prone to disturbances in triglyceride (TG) and cholesterol metabolism. This is due to the downregulation of acetyl CoA acetyltransferase 2 in the liver, which inhibits cholesterol synthesis, promotes TG synthesis, and reduces the levels of very‐low‐density lipoproteins (VLDL) and low‐density lipoprotein‐C. Consequently, there is an increased risk of developing fatty liver (Zhou et al., 2023).

Both clinical and subclinical cases of ketosis result in significant economic losses for the dairy industry, making prevention strategies crucial (Liang et al., 2017). Some studies from the United States have reported that a case of SCK can cost between US$77 and 289 (Liang et al., 2017; McArt et al., 2015), and in a Canadian study, the cost was reported at CAN$203 per case (Gohary et al., 2016). More recently, the cost for a case of SCK in the Netherlands ranged from 39 to 348 euros (Mostert et al., 2018).

This review will provide updated information on the disease's epidemiology, its connection to specific genetic traits in dairy cattle, feeding management, body condition, fertility, secondary diseases, treatment options and preventive measures at the herd level. It will also delve into the association between ketosis and abdominal adiposity, emphasizing its importance.

## 2 PHYSIOLOGIOCAL AND PATHOPHYSIOLOGICAL ASPECTS

Ketosis is a complex metabolic disease that poses challenges in its definition, as the synthesis of ketone bodies, known as ketogenesis, is actually a normal physiological process in mammals, including dairy cows. This metabolic pathway involves the convergence of carbohydrates, fats and proteins, resulting in the accumulation of acetyl Co‐A within the mitochondria of cells, particularly hepatocytes (Nelson & Cow, 2004).

The development of ketosis is influenced by the lack or depletion of normal precursors required for the Krebs cycle, particularly oxaloacetate. This occurs due to an increased rate of gluconeogenesis and the inhibition of several enzymes within the Krebs cycle caused by the substantial production of NADH^+^ during the β‐oxidation of acetylated fatty acids that enter the mitochondria. As a consequence, the accumulated acetyl Co‐A cannot undergo further oxidation within the Krebs cycle and is diverted towards the formation of ketone bodies. These ketone bodies are subsequently released into the bloodstream, serving as an energy source for specific tissues in the body, such as the heart and brain (Nelson & Cox, 2004; Ruppert & Kersten, 2024; Zhout et al., 2023).

In more detail, the production of ketone bodies primarily occurs in the liver, triggered by various factors that result in reduced glucose concentration in the bloodstream. One such factor is the increased milk production and subsequent lactose synthesis, which require glucose as a precursor. Consequently, glucagon activation in the liver inhibits lipogenesis, decreases glycogen synthesis and glycolysis rates, stimulates gluconeogenesis and glycogenolysis, enhances fatty acid oxidation, and promotes ketogenesis. Conversely, insulin, which is reduced during the catabolic state experienced by cows in the post‐partum period, exhibits opposing effects (Melendez, 2017; Nelson & Cox, 2004; Ruppert & Kersten, 2024).

The onset of ketogenesis coincides with a decline in blood glucose levels due to the heightened synthesis of lactose in the mammary gland. However, increased ketone body synthesis can also arise from the development of insulin resistance, particularly in the early post‐partum period (2–14 days after calving) (Drackley et al., 2001; De Koster & Opsomer, 2013). Additionally, during proteolysis, certain amino acids with ketogenic properties, such as leucine, lysine, tryptophan, alanine and tyrosine, undergo deamination and contribute to the formation of AcAc (Nelson & Cox, 2004).

Indeed, the term ‘bodies’ is a historical designation that has been used in reference to ketone compounds. However, it is important to note that these compounds are highly soluble in blood and body fluids, despite occasional references to them as insoluble particles. Among the ketone bodies, Ac is produced in smaller quantities compared to the others. It is exhaled and gives the characteristic odour associated with cows experiencing ketosis. AcAc and BHB are transported through the bloodstream to extrahepatic tissues. Once there, they undergo conversion into acetyl‐CoA and are oxidized in the Krebs cycle, providing essential energy for muscle, cardiac and renal cortex tissues. The brain, which primarily relies on glucose as its preferred energy source, can adapt to utilize AcAc and BHB during periods of severe negative energy balance when glucose availability is limited (Nelson & Cox, 2004; Ruppert & Kersten, 2024).

The initial step in the formation of AcAc involves the enzymatic condensation of two acetyl‐CoA molecules, facilitated by the enzyme thiolase. This process is essentially the reverse of the final step in β‐oxidation. Aceto‐acetyl‐CoA then combines with acetyl‐CoA to generate β‐hydroxy‐β‐methylglutaryl‐CoA, which undergoes cleavage to release AcAc and additional acetyl‐CoA. AcAc can be reversibly reduced to BHB through the action of BHB dehydrogenase. In small quantities, Ac is formed from AcAc, which can be decarboxylated spontaneously or with the assistance of aceto‐acetate decarboxylase (Nelson & Cox, 2004).

In extrahepatic tissues, BHB is oxidized back to AcAc with the involvement of BHB dehydrogenase. AcAc is activated by the transfer of a CoA group from succinyl‐CoA, a reaction mediated by β‐ketoacyl‐CoA transferase. The resulting aceto‐acetyl‐CoA is then cleaved by thiolase, resulting in the production of two acetyl‐CoA molecules that enter the Krebs cycle. Through this process, ketone bodies serve as an energy source, enabling the continuous oxidation of fatty acids while minimizing the oxidation of acetyl‐CoA (Ruppert & Kersten, 2024).

**FIGURE 1:**
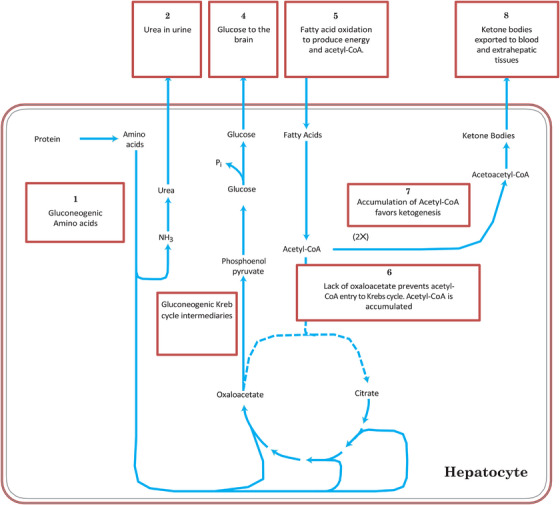
Main biochemical steps of carbohydrate, fat and protein metabolism that regulate ketogenesis in mammals. *Source*: Nelson and Cox (2004).

**FIGURE 2:**
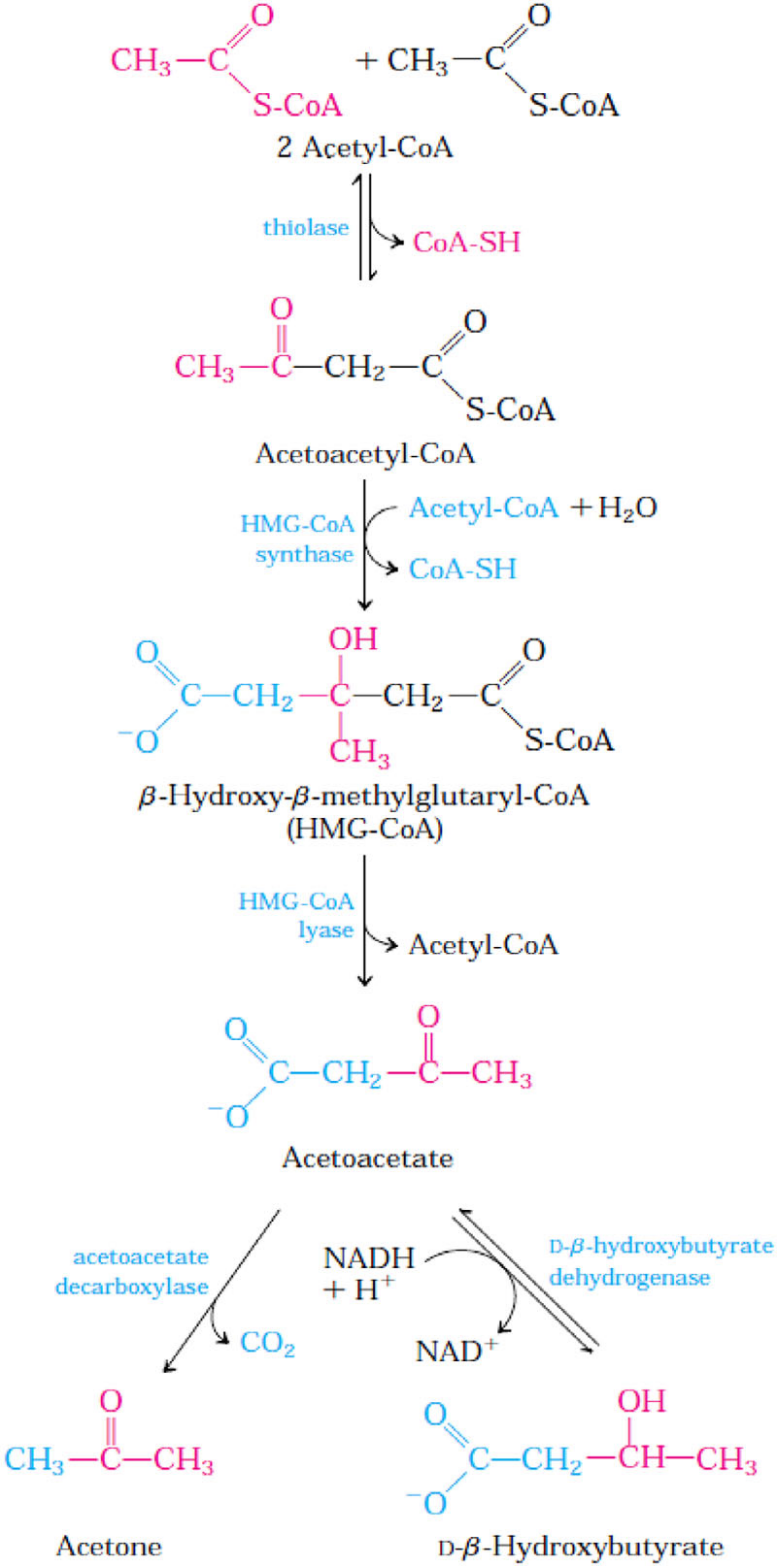
Enzymes and compounds involved in ketogenesis. *Source*: Nelson and Cox (2004).

When intermediates of the Krebs cycle are redirected towards glucose production, the oxidation of these intermediates slows down. Moreover, the liver possesses a limited quantity of coenzyme A, which becomes a limiting factor for both β‐oxidation and the Krebs cycle (Nelson & Cox, 2004; Ruppert & Kersten, 2024). A summary of the hormones, enzymes, compounds and cytokines/adipokines involved in ketogenesis can be found in Table 1 and Figures 1 and 2.

**TABLE 1:** Hormonal, enzymatic and biochemical control of ketogenesis.

Compound	Effect	Enzyme/receptor
**Insulin** Decreases ketogenesis	↑ Glucose entry into the cell (muscle and adipocytes) ↑ Glucose entry into the cell (liver) ↑ Glycogen synthesis in liver and muscle ↓ Glycogenolysis (liver and muscle) ↑ Glycolysis and acetyl‐CoA (liver and muscle) ↑ Fatty acid synthesis (liver) ↑ Triglyceride synthesis (adipose tissue)	↑ GLUT4 ↑ Glucokinase ↑ Glycogen synthetase ↓ Glycogen phosphorylase ↑ PFK‐1, ↑ pyruvate dehydrogenase ↑ Acetyl‐CoA carboxylase ↑ Lipoprotein lipase
**Glucagon** Increases Ketogenesis	↑ Hepatic glycogenolysis ↓ Hepatic glycogen synthesis ↓ Hepatic glycolysis ↑ Hepatic gluconeogenesis en hígado ↑ Lipolysis ↑ Ketogenesis	↑ Glycogen phosphorylase ↓ Glycogen synthetase ↓ PFK‐1 ↑ FBPase‐2, ↓ pyruvate kinase, ↑ PEP carboxykinase ↑ Triacylglycerol lipase, phosphorylation perilipin ↑ Acetyl‐CoA carboxylase
Epinephrine Increases ketogenesis	↑ Heart rate ↑ Blood pressure ↑ Respiratory tract dilation ↑ Glycogenolysis in liver and muscle ↓ Glycogen synthesis in liver and muscle ↑ Glycolysis in muscle ↑ Glyconeogenesis in liver ↑ Glucagon, lipolysis and ketogenesis ↓ Insulin	↑ Perilipin phosphorylation
**Adiponectin** Increases ketogenesis	↑ Fatty acid entry in muscle ↑ Beta‐oxidation ↑ Glucose entry in muscle ↑ Glycolysis in liver ↓ Gluconeogenesis ↓ Fatty acid synthesis	↑AMPK
**Cortisol** Increases ketogenesis	↑ degradación de proteínas en músculo ↑ gluconeogénesis en hígado ↑ lipolisis y cetogénesis ↓ Insulina	
**Fatty acids in hepatocytes**	Stimulation of PPARα in cell nucleus of hepatocytes, activating beta oxidation and ketogenesis controlling genes during negative energy balance	Superfamily of nuclear receptors PPARα: beta‐oxidation in liver PPARδ: beta‐oxidation in liver and muscle. Energy dissipation by mitochondrial uncoupling PPARγ: lipogenesis in adipose tissue and liver

In greater detail, when blood glucose levels are low (e.g. during high lactose synthesis in the mammary gland of high‐producing dairy cows), the release of glucagon and epinephrine is triggered. Glucagon binds to its receptor on the adipocyte membrane, stimulating adenylyl cyclase through a G protein, leading to the production of cAMP. This activates phosphokinase A, which phosphorylates hormone‐sensitive lipase and perilipin molecules on the surface of lipid droplets. Phosphorylation of perilipin allows hormone‐sensitive lipase to access the surface of the lipid droplet, where it hydrolyses TG into free fatty acids. These fatty acids leave the adipocyte, bind to serum albumin in the blood, and are transported throughout the body. They are then released from albumin and taken up by various cells, including hepatocytes, through specific fatty acid transporters. In these cells, fatty acids are oxidized to produce CO_2_, and the energy generated from oxidation is stored in ATP, which fulfils the energy requirements for milk production (Melendez & Risco, 2021; Nelson & Cox, 2004).

Most fatty acids with 16 or more carbon atoms, which constitute the majority of fatty acids released from adipose tissue, cannot directly pass‐through mitochondrial membranes. Instead, they undergo three enzymatic reactions associated with the carnitine shuttle. The first reaction occurs in the outer mitochondrial membrane and involves a family of isozymes called acyl‐CoA synthetases. These enzymes catalyse the formation of a thioester linkage between the carboxyl group of the fatty acid and the thiol group of coenzyme A, resulting in the production of fatty acyl CoA and the cleavage of ATP to AMP and phosphate. Similar to acetyl‐CoA, fatty acyl CoA is a high‐energy compound that can be transported into the mitochondrion from the cytosolic side of the outer mitochondrial membrane, where it can be oxidized to generate ATP or used for the synthesis of membrane lipids within the cytosol (Nelson & Cox, 2004).

Fatty acids destined for mitochondrial oxidation are temporarily attached to the hydroxyl group of carnitine in a process called transesterification, which constitutes the second reaction of the carnitine shuttle. This transesterification is catalysed by carnitine acyltransferase I, located in the outer membrane of mitochondria. The resulting fatty acyl carnitine ester enters the mitochondrial matrix through facilitated diffusion, enabled by the acyl‐carnitine/carnitine transporter present in the inner mitochondrial membrane. In the third and final step of the carnitine shuttle, the fatty acyl group is enzymatically transferred from carnitine to intramitochondrial coenzyme A by carnitine acyltransferase II. This specific isozyme, situated on the inner face of the inner mitochondrial membrane, regenerates fatty acyl CoA and releases it, along with free carnitine, into the mitochondrial matrix. Carnitine then re‐enters the intermembrane space via the acyl‐carnitine/carnitine transporter. This three‐step process of transferring fatty acids into the mitochondrion, involving esterification to CoA, transesterification to carnitine and subsequent transport and reconversion to CoA, connects two distinct pools of coenzyme A present in the cytosol and mitochondria, each serving different functions. Coenzyme A in the mitochondrial matrix is primarily utilized in the oxidative degradation of pyruvate, fatty acids and certain amino acids, whereas cytosolic coenzyme A is involved in the biosynthesis of fatty acids, membrane lipid synthesis, or can be transported into the mitochondrial matrix for oxidation and ATP production. The carnitine‐mediated entry process serves as the rate‐limiting step for fatty acid oxidation in mitochondria, thus playing a crucial role in the regulation of energy metabolism (Nelson & Cox, 2004; Ruppert & Kersten, 2024).

Because fatty acid oxidation is tightly regulated and inhibited by malonyl‐CoA (the initial intermediate in the cytosolic biosynthesis of long‐chain fatty acids from acetyl‐CoA), its concentration increases when the animal is well‐nourished, primarily with carbohydrates. When there is an excess of glucose that cannot be oxidized or stored as glycogen, it is converted into fatty acids in the cytosol for storage as TG. The inhibition of carnitine acyltransferase I by malonyl‐CoA ensures that the oxidation of fatty acids is suppressed when the liver is sufficiently fuelled with glucose and actively synthesizing TG from the excess glucose. However, when intermediates of the Krebs cycle are diverted for glucose synthesis through gluconeogenesis or when the amount of coenzyme A is limited in the liver, as observed in high‐producing cows, the levels of malonyl‐CoA decrease. This relief of inhibition on carnitine acyltransferase I allows fatty acids to enter mitochondria for degradation into acetyl‐CoA. However, acetyl‐CoA cannot directly enter the citric acid cycle because the cycle intermediates have already been utilized, leading to the redirection of acetyl‐CoA towards ketone body formation (Nelson & Cox, 2004).

In a recent study, it was reported using a transcriptomic approach that cows with SCK exhibited significantly lower levels of acetyl CoA acetyltransferase 2 (thiolase). Additionally, cows with SCK showed a significant increase in the expression of peroxisome proliferator‐activated receptor γ and sterol regulator element binding protein 1c, while having significantly lower expression of peroxisome proliferator‐activated receptor α, carnitine palmitoyl‐transferase 1A, sterol regulatory element binding transcription factor 2 and 3‐hydroxy‐3‐methylglutaryl‐CoA reductase. Furthermore, cows with SCK displayed higher concentrations of liver TG and significantly lower levels of plasma total cholesterol and free cholesterol content. Moreover, these cows exhibited increased expression of perilipin‐2, decreased expression of apolipoprotein B, and reduced plasma concentration of VLDL and LDL‐C, all of which indicated the accumulation of TG in hepatocytes. These findings suggest that the downregulation of acetyl CoA acetyltransferase 2 in the liver promotes TG accumulation and inhibits cholesterol synthesis, thereby indicating a higher propensity for fatty liver in dairy cows (Zhou et al., 2023).

In another recent study, cows with BHB >1.2 mmol/L had increased levels of biomarkers of pain, inflammation, including lipopolysaccharide‐binding protein, haptoglobin, serum amyloid A and proinflammatory cytokines IL‐4, MCP‐1, MIP‐1α and TNFα. In addition, 72.2% of cows with hyperketonemia had endotoxemia and higher circulating bacterial DNA when compared to a control group (Chirivi et al., 2023). This study highlights and confirms the importance of the pro‐inflammatory status and the occurrence of infectious processes and endotoxemia in cows with high concentrations of BHB in blood.

## 3 EPIDEMIOLOGY

SCK has been defined using different criteria, with ROC curve analyses determining various cut‐off values for blood BHB concentration. The cut‐off range has been established between 1.0 and 1.4 mmol/L. Canadian studies have defined the cut‐off at 1.4 mmol/L, whereas US studies have set it at 1.2 mmol/L (Carrier et al., 2004; Duffield et al., 2009; McArt et al., 2012; Ospina et al., 2013; Shin et al., 2015). In New Zealand grazing cattle, the cut‐off value was determined to be 1.2 mmol/L (Compton et al., 2015). Meanwhile, in a study of grazing cows in southern Chile, the blood BHB cut‐off value to define cows with SCK was 1.1 mmol/L (Melendez, Chacon et al., 2020).

A recent study involving 3758 cows from 589 herds across 10 countries, an analysis of 9143 milk samples from 7 dairy breeds was conducted using mid‐infrared spectrometry (MIR). The aim was to identify biomarkers related to energy deficit, ketosis, mastitis and fertility. Several compounds, including citrate, isocitrate, glucose‐6‐phosphate, free glucose, BHB, Ac and progesterone, were evaluated in this study. The results indicated that the MIR spectra of milk could be used to quantitatively screen citrate as a biomarker for energy deficit. Additionally, BHB concentrations were found to be discriminative and could serve as biomarkers for ketosis. A cut‐off value of 200 µmol/L for milk BHB concentration was calculated for SCK, with a sensitivity of 88%, specificity of 92% and accuracy of 92% (Grelet et al., 2024).

Several other epidemiological studies have provided estimates of the prevalence of SCK in different regions. In the United States, the prevalence has been reported to range from 7.6% to 33.1% (Carrier et al., 2004; Meléndez et al., 2006a; Shire et al., 2013). In Europe, the prevalence ranged from 5.1% to 20.5% (Iwersen et al., 2009; Jorritsma et al., 1998). Grazing herds in Argentina and New Zealand have shown prevalences of 10.3% and 17.9%, respectively (Compton et al., 2014; Garro et al., 2014). In the Middle East, the prevalence has been reported between 3.8% and 68% (Al‐Rawashdeh, 1999; Asl et al., 2011). In a recent study conducted in Chile, the prevalence of SCK in grazing cows was determined using a cut‐off value of 1.1 mmol/L of BHB in blood. It was found that the prevalence at 7 days post‐partum was 22.2%. The prevalence was higher in cows calving in spring (27.0%) compared to those calving in autumn (10.3%). Furthermore, the prevalence was higher in multiparous cows (24.6%) compared to primiparous cows (15.1%). The seasonal difference observed was dependent on the lactation number, as the prevalence was higher in multiparous cows with spring parturitions (32.0%) compared to autumn (10.1%), whereas the prevalence in primiparous cows did not show significant differences between spring (15.4%) and autumn (12.5%) (Melendez, Chacon et al., 2020).

In a recent study conducted in Belgium and the Netherlands, the incidence of SCK was evaluated using an automated real‐time data system called the DeLaval Herd Navigator. Milk samples were collected from individual cows between 20 and 60 days post‐partum, and milk BHB levels were measured. The study used three different cut‐off values (0.08, 0.10 and 0.15 mmol/L) to identify cases of SCK. The results showed that the incidence of SCK varied depending on the cut‐off value used. For the cut‐off value of 0.08 mmol/L, the incidence was reported as 76.7% (95% CI 53.8–93.9). When the cut‐off value was set at 0.10 mmol/L, the incidence decreased to 52.0% (95% CI 28.8–78.0). Finally, for the cut‐off value of 0.15 mmol/L, the incidence further decreased to 30.5% (95% CI 11.1–60.9) (De Jong et al., 2023).

In recent years, there has been a growing evidence of the high incidence of SCK in dairy cattle, particularly in the United States. Studies have shown that the highest incidence of SCK, defined as blood BHB levels exceeding 1.2 mmol/L, occurs between 3 and 7 days post‐partum. Incidence values greater than 20% have been reported at day 4 and 5 post‐partum (McArt et al., 2012; Ospina et al., 2013).

Cows diagnosed with SCK face various challenges and risks compared to healthy cows. They have a higher risk of developing DA compared to healthy cows (6.5% vs. 0.3%, respectively) and are more likely to be culled early from the herd (5.4% vs. 1.8%, respectively). Furthermore, cows with SCK exhibit a lower conception rate at first service (40% vs. 35.1%) and a lower daily milk yield (33.9 vs. 35.1 kg/day) compared to healthy cows (McArt et al., 2012).

Interestingly, cows diagnosed with SCK during the first 7 days post‐partum have been found to have significantly higher odds of experiencing DA (6.1 times greater), being culled early from the herd (4.5 times higher odds), having lower odds of conceiving at the first service (0.7 times odds), and producing less milk per day (2.1 kg less) compared to cows diagnosed with SCK during the second week post‐partum (McArt et al., 2012).

In another recent study conducted in Japan, SCK was defined as blood BHB levels ≥1.2 mM. The study reported a SCK prevalence of 20.2% within the first 14 days post‐partum, with a tendency for higher occurrence compared to SCK between 15 and 80 days post‐partum (16.5%) (Chisato et al., 2023). These findings emphasize the importance of early diagnosis and timely intervention for SCK within the first week post‐partum. Early treatment or prevention strategies can help mitigate associated losses and improve cow health and productivity.

There are several risk factors that have been identified for SCK in dairy cattle. High milk production, both in confined and grazing cattle, has been associated with an increased incidence of ketosis (McArt et al., 2012; Melendez, 2017; Melendez & Risco, 2021). Additionally, a higher body condition score (BCS) during the prepartum period and at calving, as well as a higher incidence of post‐partum diseases, have been linked to an increased risk of SCK (McArt et al., 2012; Melendez, 2017; Melendez & Risco, 2021; Ospina et al., 2013).

The breed of the cow has also been identified as a risk factor for ketosis. Jersey cows have been found to be more likely to develop ketosis compared to primiparous Holstein cows and multiparous Holstein cows (Tatone et al., 2017). In Sweden, the Red and White Swedish breeds were reported to have higher concentrations of AcAc in milk compared to the Swedish Friesian breed (Andersson & Emanuelson, 1985). However, other studies have not found a consistent relationship between dairy breed and the occurrence of ketosis (Berge & Vertenten, 2014).

Milk production plays a significant role in the incidence of ketosis in dairy cattle, although the direction of this relationship can vary. In a study, it was observed that the relationship between milk production and ketosis depends on the lactation number of the cows. Primiparous cows with higher production above the herd median were actually less likely to develop ketosis compared to primiparous cows in the lowest quartile of the herd median. However, in multiparous cows, the lowest quartile average had a protective effect against ketosis compared to animals in herds with higher average production (Tatone et al., 2017). Another study conducted in Japan also reported elevated BHB concentrations in dairy cows with higher production (Sofyan et al., 2017). However, the average milk production of the herd may also be affected by the prevalence of ketosis, and there may be a reverse causality relationship. In other words, a higher prevalence of ketosis within the herd might result in a decrease in the average milk production of the herd but could be associated with higher milk production from individual cows. Other studies have also found a higher risk of ketosis in cows producing more milk, both in grazing and confined cattle (Garro et al., 2014; Liang et al., 2017; Van Holder et al., 2015). However, a meta‐analysis concluded that the association between ketosis and milk production is unclear, and it can be both positive and negative depending on the timing of ketosis diagnosis and the lactation number of the cow (Raboisson et al., 2014). Additionally, greater cumulative milk production from the previous lactation has been associated with higher odds of developing ketosis in the current lactation (Rasmussen et al., 1999). However, some studies have found no association between previous lactation production and the risk of developing ketosis during the early post‐partum period of the current lactation (Gordon, 2013).

The length of the dry period has also been identified as a risk factor for ketosis. As the length of the dry period increases, the odds of developing ketosis also increase. Similarly, a calving interval of 13 to 15 months has been associated with higher odds of developing ketosis compared to a calving interval of 12 months, and cows with a calving interval exceeding 15 months have even higher risk (Tatone et al., 2017).

Furthermore, the age at first calving has been found to be a risk factor for ketosis. Primiparous cows with their age at first calving equal to or greater than 25 months had higher odds of developing ketosis compared to primiparous cows with their age at first calving below 25 months (Tatone et al., 2017). A similar association was reported by Gordon (2013), who found that cows with their first calving occurring after 24 months had an increased risk of ketosis compared to cows calving at a younger age. These identified risk factors highlight the complexity of SCK and the importance of considering multiple factors in its prevention and management strategies.

The association between BCS and the risk of ketosis in dairy cattle is significant. Higher prepartum BCS has been consistently linked to a higher incidence of post‐partum ketosis (Gillund et al., 2001; McArt et al., 2013). A comprehensive study conducted in Argentina, covering multiple dairy herds, shed light on the crucial role of BCS dynamics during the dry period in explaining various variables such as disease prevalence and milk production (Melendez, Bargo et al., 2020). The study's first important finding revealed that over 40% of the cows experienced BCS loss between drying‐off and calving. Moreover, it highlighted that BCS losses during the early dry period were less detrimental than those occurring during the prepartum period. Cows that maintained or gained BCS throughout the entire dry period had a lower likelihood of developing retained foetal membranes, clinical mastitis and metritis compared to cows that maintained BCS during the early dry period but experienced BCS loss during the prepartum period. Interestingly, these cows also exhibited a reduced likelihood of developing retained foetal membranes and clinical mastitis compared to cows that lost BCS throughout the entire dry period. Additionally, they tended to have lower levels of SCK compared to cows that only lost BCS during the prepartum period. Furthermore, cows that maintained or gained BCS throughout the dry period had higher milk production compared to cows that lost BCS only during the prepartum period or throughout the entire dry period.

Notably, cows with greater BCS have been associated with increased abdominal adiposity, as highlighted in a study by Melendez et al. (2023). Abdominal fat exhibits higher reactivity with an elevated rate of lipolysis, and it also possesses a more pro‐inflammatory nature (Tchkonia et al., 2013). This suggests that cows with greater abdominal adiposity are more prone to developing ketosis, fatty liver, DA and other associated diseases. In summary, proper management of BCS throughout the entire dry period, with a particular focus on avoiding BCS losses, especially during the prepartum period, is of utmost importance. This emphasizes the significance of maintaining optimal BCS to reduce the risk of ketosis and related health issues in dairy cattle. Additionally, the association between high BCS and increased abdominal adiposity further underscores the need for proactive measures to mitigate the occurrence of ketosis and its associated complications.

The association between SCK and other diseases in dairy cattle is well‐established. Cows with SCK have been found to be at a higher risk of developing various health disorders. For instance, they are 3.33 times more likely to develop DA, 1.92 times more likely to be removed from the herd or die, 1.75 times more likely to develop metritis, 1.52 times more likely to experience retained foetal membranes, 1.61 times more likely to develop clinical mastitis and 2.01 times more likely to develop lameness (McArt et al., 2012).

The negative impact of SCK extends beyond health. Fertility is also affected, with cows exhibiting lower probabilities of conception at first insemination and increased average time to pregnancy (Rutherford et al., 2016; Shin et al., 2015). This highlights the importance of addressing SCK to maintain optimal reproductive performance in dairy herds. Grazing dairy cattle are not exempt from the association between SCK and other diseases. Studies conducted on grazing cows have shown that those with SCK have a 1.8 times greater risk of presenting other diseases compared to cows without SCK (Melendez, Chacon et al., 2020). Interestingly, despite the higher prevalence of SCK in spring‐calving multiparous cows, these cows managed on pasture were able to produce more milk and maintain normal fertility, suggesting the influence of management practices on mitigating the negative effects of SCK (Melendez, Chacon et al., 2020). This information underscores the importance of addressing SCK not only for its direct impact on cow health but also for its potential implications on fertility and overall productivity in dairy herds. Effective management strategies, including proper nutrition, monitoring and prompt intervention, can help reduce the incidence of SCK and its associated diseases, ultimately leading to improved herd health and performance.

## 4 DIAGNOSIS

The diagnosis of ketosis in dairy cattle relies on measuring the levels of ketone bodies, particularly AcAc and BHB, in various body fluids such as urine, milk, serum, plasma or blood (Iwersen et al., 2009; Melendez, 2017; Ospina et al., 2013). Different methodologies exist for each fluid, each with its own characteristics in terms of sensitivity, specificity, positive predictive value and negative predictive value. Table 2 provides a summary of these values for different ketone body measurement methods in dairy cattle. The studied tests include Precision Xtra (Abbott Diabetes Care), Ketostix strip (Bayer) and Ketolac (Biolab).

**TABLE 2:** Sensitivity, specificity, positive and negative predictive values of different methods for measuring ketone bodies in dairy cattle (Iwersen et al., 2009).

Test	Fluid	Cut‐off (µmol/L)	Se^a^% (CI 95%)e	Spb% (CI 95%)	PV (+)c (CI 95%)	PV (−)d (CI 95%)
Precision Xtra	Blood	1.400	100 (69–100)	100 (94–100)	100 (69–100)	100 (98–100)
Precision Xtra	Milk	200	60 (26–88)	89 (83–93)	22 (9–42)	98 (94–99)
Precision Xtra	Milk	300	40 (12–74)	97 (94–99)	44 (14–79)	97 (93–99)
Ketolac	Milk	100	90 (56–100)	94 (90–97)	45 (23–68)	99 (97–100)
Ketolac	Milk	200	30 (7–65)	98 (95–100)	50 (12–88)	96 (93–99)
Precision Xtra	Urine	1.000	100 (66–100)	25 (19–32)	6 (3–14)	100 (92–100)
Precision Xtra	Urine	2.000	67 (30–93)	86 (80–91)	19 (7–37)	98 (95–100)
Precision Xtra	Urine	3.000	56 (21–86)	98 (94–99)	56 (21–86)	98 (94–99)
Ketostix	Urine	500	78 (40–98)	92 (87–96)	33 (15–57)	99 (96–100)
Ketostix	Urine	1.500	67 (30–93)	97 (94–99)	36 (23–83)	100 (95–100)
Ketostix	Urine	4.000	67 (30–93)	100 (98–100)	100 (54–100)	98 (95–100)
Ketostix	Urine	8.000	44 (14–79)	100 (98–100)	100 (40–100)	97 (94–99)
Ketostix	Urine	1.000	22 (3–60)	100 (98–100)	100 (16–100)	98 (92–98)

^a^Sensitivity.

^b^Specificity.

^c^Positive predictive value.

^d^Negative predictive value.

^e^95% Confidence interval.

To establish a gold standard for comparison, the measurement of BHB in serum was used, employing a colorimetric enzymatic system known as the Ranbut D‐3‐hydroxybutyrate kit from Randox Laboratories (Antrim, UK). This measurement was performed on an automated chemistry analyser, specifically the Olympus AU 400 (Olympus).

During the last time, milk analysis using Fourier transform infrared spectroscopy has been reported as a method allowing for sampling numerous cows simultaneously, which has a moderate accuracy for the diagnosis of SCK (BHB ≥1.2 mmol/L) (Walleser et al., 2023)

These standardized methods and comparisons allow for accurate and reliable diagnosis of ketosis in dairy cattle, aiding in effective management and intervention strategies. It is essential to choose the appropriate measurement method based on the specific requirements and characteristics of the diagnostic situation to ensure optimal results and informed decision‐making.

## 5 TREATMENT AND PREVENTION

Preventing and effectively treating ketosis in dairy herds is crucial due to the associated costs and negative consequences. Managing and controlling the risk factors linked to this metabolic condition has become essential (Melendez & Risco, 2021). Although it may be challenging to completely eliminate ketosis, especially subclinical cases, the treatment of ketosis should be effective in mitigating its adverse effects, such as reduced milk production, infertility and secondary diseases like DA, which is the costliest reported disease in dairy cattle, averaging at US$650 per case (Liang et al., 2017).

Recent studies have explored various treatment strategies for ketosis. These include using dexamethasone as an adjunct therapy to propylene glycol, a product combining butophosphane and cyanocobalamin, or insulin in combination with propylene glycol, as well as daily injections of vitamin B_12_ compared to a control group (Gordon et al., 2013; Tatone, Gordon et al., 2016; Tatone, Duffield et al., 2016; Weerathilake et al., 2019). However, the results have not been consistent, and studies evaluating adjunctive treatments to propylene glycol did not demonstrate a significant improvement in post‐partum health or reproductive performance compared to treatment with propylene glycol alone (Jeong et al., 2018). Therefore, the primary treatment for ketosis continues to be the use of propylene glycol, either in liquid form for oral treatment or as a powder mixed into the total ration (Ospina et al., 2013; McArt et al., 2011). Recent research supports the effectiveness of propylene glycol as the sole treatment without the need for additional therapies like intravenous dextrose (Capel et al., 2021). This study found that the addition of intravenous dextrose did not enhance the resolution of SCK or improve average daily milk production compared to treatment with propylene glycol alone. Moreover, the administration of dextrose can be labour‐intensive and may carry the risk of jugular thrombophlebitis from extravascular administration. Hence, propylene glycol alone remains the recommended treatment for clinical and SCK, as supported by previous studies highlighting its positive effects in resolving the metabolic condition, preventing clinical ketosis, and improving milk production in early lactation (McArt et al., 2011). In conclusion, the effective management and prevention of ketosis in dairy herds, along with the use of propylene glycol as the primary treatment, play key roles in minimizing the economic and health impacts associated with this metabolic condition.

A recent study offers interesting insights into therapeutical approaches for managing lipolysis in cows with ketosis. By targeting both the hormonal and inflammatory pathways of lipolysis, the researchers explored the use of niacin and flunixin meglumine as potential treatments. The group treated with propylene glycol + niacin + flunixin meglumine showed promising results, with a higher percentage of cows achieving normoketonemia compared to the other treatment groups. These cows were also more likely to have normal levels of BHB and exhibited lower concentrations of BHB, acute phase proteins and NEFA. Additionally, their blood glucose and insulin concentrations were higher, indicating improved metabolic status (Chirivi et al., 2023). Undoubtedly, this study provides a valuable treatment approach that complements the use of propylene glycol alone by incorporating niacin and flunixin meglumine to control lipolysis in cows with ketosis.

On the other hand, preventing ketosis requires a comprehensive approach that includes a rational and strategic feeding programme as well as appropriate cow comfort and management. It is essential to provide well‐balanced diets in terms of energy, protein, vitamins and minerals. Maintaining an optimal BCS throughout the dry and transition period is crucial, as both excessive BCS and abdominal adiposity at calving and BCS losses during the dry period can increase the risk of peripartum disorders.

To prevent ketosis, including gluconeogenic precursors in the diet can be beneficial. These precursors promote hepatic gluconeogenesis, increase circulating insulin levels, and reduce hepatic TG accumulation and NEFA mobilization. Additives, such as glycerol, calcium propionate and propylene glycol, have proven successful in preventing and treating ketosis in dairy cows. It is recommended to offer these additives as top‐dressed on the entire ration at the feed‐bunk rather than mixing them with all ingredients. The routine use of supplements like propylene glycol should be based on the incidence of ketosis in the herd (Melendez & Risco, 2021; Ospina et al., 2013).

Sodium monensin is another effective nutritional additive for preventing bovine ketosis. It can be administered in either powder form or as a slow‐release ruminal bolus. Monensin promotes the production of propionic acid in the rumen, which is a key gluconeogenic precursor in ruminants. This helps reduce the incidence of ketosis and related disorders while improving milk production and fertility. Additionally, monensin can be used during the dry period to improve body reserves in cows with low BCS, ensuring they calve with adequate BCS (Duffield et al., 2012; Melendez, Goff et al., 2006; Melendez, Gonzalez et al., 2006; Melendez et al., 2007).

In conclusion, a combination of therapeutical approaches and preventive measures, alongside a well‐designed feeding programme and proper cow‐comfort and management, can effectively address ketosis in dairy herds.

## 6 PATHOPHYSIOLOGICAL HYPOTHESIS OF CURRENT BOVINE KETOSIS

The occurrence of SCK has undergone a shift in recent years. Previously, cases were more commonly observed between 14 and 21 days post‐partum, whereas now they occur with greater frequency between 3 and 5 days post‐partum. This change can be attributed to the intense genetic selection pressure for milk production. Nowadays, cows start producing substantial amounts of milk shortly after calving, resulting in peak production being reached between 20 and 30 days in milk, as opposed to the previous norm of 45–60 days in milk (Kerwin et al., 2023; McArt et al., 2012; Melendez & Risco 2021).

To achieve this early high milk yield, dairy cows have had to undergo metabolic modifications, depositing more adipose tissue at the abdominal level. This abdominal adipose tissue serves as a readily mobilized energy source compared to subcutaneous fat. The active lipolysis of abdominal adipose tissue leads to the release of NEFA into the portal venous system, which then swiftly reaches the liver. These fatty acids can be rapidly metabolized through β‐oxidation to generate energy efficiently. Alternatively, the liver can export these fatty acids in the form of VLDL, which can be directed to the mammary gland and incorporated into milk fat as fatty acids with 16 or more carbons. Fatty acids with 16 or fewer carbons are synthesized within the mammary gland through the ‘de novo’ synthesis process, starting from butyrate molecules (4 carbons) from the rumen or liver and coupling them with acetate molecules (2 carbons) to elongate the fatty acid chain up to 16 carbons (Contreras et al., 2018; Drackley et al., 2001; Nelson & Cox, 2004; Rupert & Kersten, 2024).

Abdominal adiposity has evolved alongside the genetic progress of milk production. It is important to note that both normal and excessive abdominal adiposity, coupled with severe lipolysis resulting from factors such as poor nutritional and environmental management, can trigger significant stress. This stress can lead to a pro‐inflammatory state, oxidative stress, excessive production of ketone bodies and initial hepatic fat accumulation, eventually leading to the development of fatty liver within the first week post‐partum (Chirivi et al., 2022, 2023; Contreras et al., 2018; Kerwin et al., 2023; Melendez & Risco 2021; Pinedo & Melendez, 2022). Furthermore, this condition can facilitate the entry of bacteria and/or endotoxins into the bloodstream, contributing to a pro‐inflammatory vicious cycle. In fact, endotoxin LPS has been shown to increase lipolysis by 70% compared to normal cows (Chirivi et al., 2022).

Excessive abdominal adiposity can trigger severe lipolysis through two primary pathways: the canonical pathway and the inflammatory pathway (Chirivi et al., 2023). In the canonical pathway, pro‐lipolytic hormones and peptides, such as catecholamines and growth hormone, activate adipocyte membrane receptors, leading to increased adenyl cyclase activity and the conversion of ATP to cAMP. This, in turn, activates protein kinase A, ultimately triggering hormone‐sensitive lipase (Chirivi et al., 2023; De Koster et al., 2018). The inflammatory pathway, on the other hand, involves the activation of inflammatory signalling cascades, such as MAPK and NFκB, which stimulate protein kinase C. This activation of protein kinase C also leads to the activation of hormone‐sensitive lipase (Chirivi et al., 2022). These altered metabolic pathways can contribute to the development of fatty liver and other associated conditions. Additionally, certain genes have been identified that are associated with the development of ketosis and fatty liver, as well as the regulation of abdominal fat deposition. These genes, known as ‘pleiotropic genes’, have the ability to control multiple characteristics simultaneously. Hence, it becomes apparent why cows that produce large quantities of milk early in lactation are also prone to marked ketonemia, fatty liver, DA and more severe pro‐inflammatory processes (Chirivi et al., 2023; Melendez et al., 2019; Novo et al., 2022). In addition, a genome‐wide association signal analysis among prevalent health conditions (mastitis, ketosis, DA, hypocalcaemia and metritis) was conducted, and 4 modules were genetically linked to overall ketosis and post‐partum ketosis. Furthermore, five candidate genes for ketosis were identified (Yan et al., 2020). These findings elucidated strong links between genetic factors associated with abdominal adiposity, ketosis and DA in Holstein cows.

In conclusion, SCK remains a prevalent disease with various risk factors and associations with other conditions. Its highest incidence occurs early in lactation and is linked to a genetic predisposition and a high degree of abdominal adiposity, fatty liver and DA. Therefore, prevention is crucial, irrespective of successful treatment options for clinical cases involving propylene glycol‐based products.

## AUTHOR CONTRIBUTIONS

**Pedro Melendez**: Conceptualization; supervision; validation; writing – original draft; writing – review and editing. **Manuel Vizcaino Serrano**: Conceptualization; validation; writing – original draft; writing – review and editing.

## CONFLICT OF INTEREST STATEMENT

The authors declare no conflicts of interest.

## FUNDING INFORMATION

None.

## ETHICS STATEMENT

None.

### PEER REVIEW

The peer review history for this article is available at https://www.webofscience.com/api/gateway/wos/peer‐review/10.1002/vms3.1525.

## Data Availability

None.
